# Rotavirus Infection as a Contributor to Early-Onset Type 1 Diabetes: Review and Recommendations

**DOI:** 10.3390/v18070727

**Published:** 2026-06-30

**Authors:** Mary A. M. Rogers, Scott O. Rogers

**Affiliations:** 1 Department of Internal Medicine, University of Michigan, Ann Arbor, MI 48109, USA; 2 Department of Biological Sciences, Bowling Green State University, Bowling Green, OH 43403, USA; srogers@bgsu.edu

**Keywords:** rotavirus infections, rotavirus vaccines, diabetes mellitus type 1, infant, child

## Abstract

Rotavirus infection is a major cause of acute gastroenteritis in children, which is characterized by fever, emesis, and diarrhea. In some children, rotaviral infection can spread beyond the gastrointestinal tract and affect the nervous system, kidneys, liver, or pancreas. There are relatively few longitudinal studies of such long-term sequalae. One area of interest has been damage to pancreatic beta islet cells, the lack of which causes type 1 diabetes mellitus. This chronic disease can be life threatening, especially in young children, and is associated with lifelong elevated risks of cardiovascular disease, neuropathy, nephropathy, and retinopathy. This narrative review summarizes the scientific evidence relevant to rotavirus infection and early-onset type 1 diabetes. The results of epidemiologic, animal, and laboratory research indicate that rotavirus infection increases the risk of type 1 diabetes in young children (<5 years of age). Rotavirus vaccination is associated with lower incidence rates; the data suggest a somewhat stronger effect with the pentavalent vaccine than the monovalent vaccine. Continued surveillance of both rotavirus infection and type 1 diabetes are necessary, considering the increases in vaccine hesitancy. The benefits of rotavirus vaccination should be discussed with parents and individuals planning to have children.

## 1. Introduction

Worldwide, rotavirus infection contributed to 120,000 deaths due to diarrhea in children younger than 5 years of age (year 2021) and 10.8 million disability-adjusted life-years [1]. For children who survive the infection, some experience long-term sequelae. Such complications include encephalitis, seizures and neurologic disease, electrolyte and metabolic imbalance, renal dysfunction, and pancreatic damage [2,3,4,5]. Over the past three decades, there have been scientific investigations of the role of rotavirus infection as a contributor to the incidence of type 1 diabetes mellitus, a chronic autoimmune disease resulting from a deficit of insulin-producing pancreatic beta cells. In this review, we summarize the evidence for one of the sequelae of rotavirus infection—early-onset type 1 diabetes.

## 2. Epidemiologic Evidence

### 2.1. Initial Studies

The connection between rotavirus gastroenteritis and pancreatic islet autoimmunity emanated from a series of studies published by Australian investigators. They reported an association between rotavirus seroconversion and specific types of antibodies known to be present in children who were genetically at high risk of developing type 1 diabetes [6]. In animal studies, they demonstrated that rotaviruses can grow and infect pancreatic islet cells [7]. Additional studies indicated that rotavirus infection resulted in pancreatic involution, decreased plasma insulin, and increased blood glucose [8]. Laboratory studies were followed by longitudinal studies in humans. In Australia, the rotavirus vaccine was introduced into the childhood vaccination schedule in 2007 [9]. Both the monovalent vaccine (Rotarix) and the pentavalent vaccine (RotaTeq) were initially available, depending upon the state or territory [9]. After rotavirus vaccine introduction, there was a 15% reduction in the incidence of type 1 diabetes in children 0–4 years of age (incidence rate ratio = 0.85; 95% CI: 0.75, 0.97) [10]. The incidence rate in children 0–4 years of age prior to introduction of the vaccine was 15.8 per 100,000 person-years and, after the vaccine was introduced, was 14.1 per 100,000 person-years [10].

These initial studies spawned further investigation. Epidemiologic studies of early-onset type 1 diabetes were focused on geographical areas where childhood type 1 diabetes was more prevalent. These included European countries and countries that historically contained a considerable proportion of the European diaspora. While there are several licensed rotavirus vaccines, evidence was available for the use of Rotarix and RotaTeq in young children [11].

### 2.2. Evidence Relevant to the Pentavalent Vaccine

**Figure 1:**
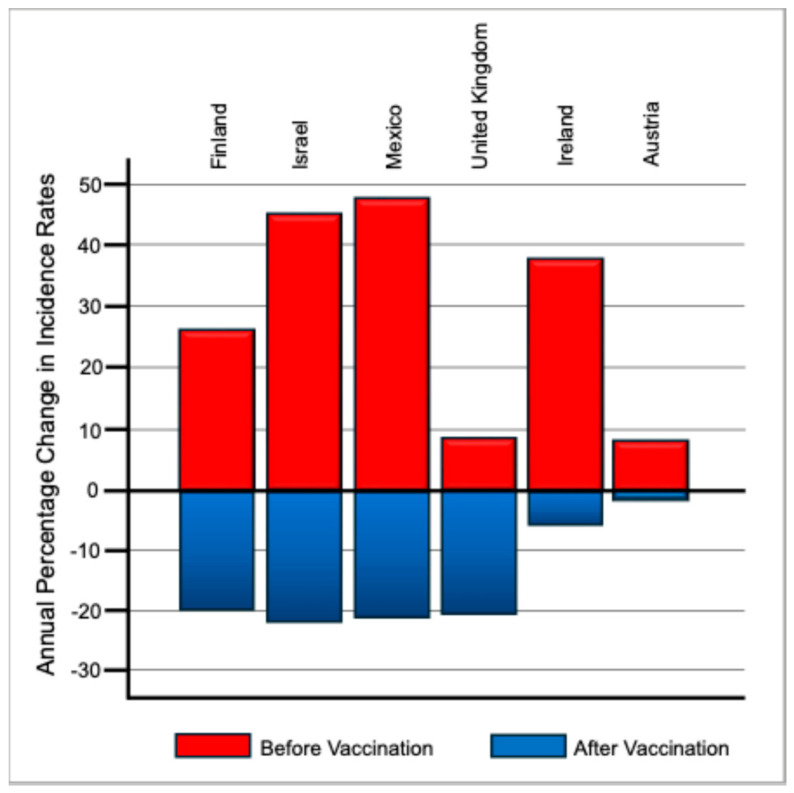
Annual percentage change in incidence rates of type 1 diabetes for children 0–4 Years of age, before and after rotavirus vaccination, by country.

Longitudinal investigations were reported from Finland, which has the highest incidence of childhood type 1 diabetes in the world [12]. The association between rotavirus infection and type 1 diabetes was examined in studies using information from their nationwide registry. The introduction of the pentavalent rotavirus vaccine (RotaTeq) to the national childhood immunization schedule occurred in 2009 [13]. There was a 23% decrease in the incidence of type 1 diabetes in children <5 years old from year 2003 (pre-vaccine) to 2018 (post-vaccine; incidence rate ratio = 0.77; *p* < 0.001) [14]. In another Finnish study, laboratory-confirmed rotavirus infection markedly declined after introduction of the rotavirus vaccine [15]. The incidence rate of type 1 diabetes in children <5 years of age significantly decreased by 21% (incidence rate ratio = 0.79; 95% CI: 0.71, 0.86) after introduction of the rotavirus vaccine [15]. A 1% reduction in rotavirus infection yielded an 8% reduction in the incidence of type 1 diabetes in the youngest children. Prior to the introduction of the rotavirus vaccine (1995–2005), there was an increase in incidence rates of type 1 diabetes in children <5 years of age (annual percentage change of 26.8%); it increased from 56.4 to 71.5 cases per 100,000 person-years [15]. After vaccine introduction, the rates decreased (−19.8%) from 67.8 to 54.4 cases per 100,000 person-years, as shown in Figure 1. It is important to note that an earlier study from Finland reported no association between rotavirus vaccination and type 1 diabetes [16]. However, their data were reanalyzed and published, showing that the percentage of children who developed type 1 diabetes was significantly (*p* = 0.002) lower in those who were vaccinated compared to the unvaccinated [17]. The risk ratio would be 0.69 (0.2573%/0.3748%), indicating a 31% reduction with vaccination and more closely aligns with the two more recent studies.

A nationwide registry for type 1 diabetes in children was also available in Israel [18]. Prior to the introduction of any rotavirus vaccines, the incidence of type 1 diabetes in children 0–4 years increased by 44.5% (Figure 1). After introduction of the pentavalent vaccine (RotaTeq) in the childhood schedule, there was a 21.7% decrease in incidence in children 0–4 years [18]. Note that the authors reported both annual percentage change and average annual percentage change (AAPC) in this study. The AAPC was 5.2% before vaccine introduction and −4.7% afterwards, which yielded a significant (*p* = 0.004) difference in slopes. The annual percentage change is shown in Figure 1 for consistency of measures across studies. Their results also showed that antibody positivity to glutamic acid decarboxylase 65 kDa isoform (GAD65), a biomarker of type 1 diabetes, was 7.5% in mothers during the pre-vaccine period compared to 1.9% after the rotavirus vaccine was introduced [18]. Cord blood results were similar (from 9.3% pre-vaccine to 1% post-vaccine). In another Israeli study, the authors found that insulin use increased in young children (<5 years) prior to rotavirus vaccination (2002–2007) and significantly decreased after RotaTeq was introduced (2011–2018) with *p* = 0.002 for the difference [19].

In Finland and Israel, results were available relevant to the use of the pentavalent vaccine. In both countries, the results demonstrated a significant difference. Additional evidence came from a large longitudinal study from the United States in which 1.47 million infants were included (years 2001–2017) [20]. Individual-level data were available. Children were followed from birth with continuous insurance coverage throughout the follow-up period to determine vaccine usage (date of administration and type of vaccine used), disease diagnoses, insulin use, and hospitalizations. Most of the infants in the study (83.3%) received the pentavalent vaccine series (RotaTeq). The children who completed the RotaTeq series had a 37% lower risk of type 1 diabetes compared to children who received no rotavirus vaccine (95% CI: 22%, 50%) [20].

In Mexico, the monovalent rotavirus vaccine (Rotarix) was used from 2007 to 2011 and the pentavalent rotavirus vaccine (RotaTeq) was introduced to the childhood vaccine schedule in 2011 [21]. In the pre-vaccine period (2001–2005), the incidence of type 1 diabetes in children <5 years increased by 47.5% [22]. During the post-vaccine period, the incidence decreased (annual percentage change = −20.8%), as shown in Figure 1. Incidence rates of type 1 diabetes in children <5 years were at their lowest after 2011 which coincided with the introduction of the pentavalent vaccine [22].

### 2.3. Evidence Relevant to the Monovalent Vaccine

Information was available from the United Kingdom, where the monovalent rotavirus vaccine (Rotarix) was introduced into the childhood immunization schedule in 2013 [23]. The incidence of type 1 diabetes in children 0–4 years of age increased (annual percentage change = 9.0%) prior to introduction of the rotavirus vaccine; the rates increased from 15.93 to 17.37 per 100,000 person-years [24]. After the vaccine was introduced, the incidence decreased (annual percentage change = −20.6%, from 17.37 to 13.80 per 100,000 person-years), as shown in Figure 1. The risk ratio comparing post-vaccine (2013–2022) to pre-vaccine (2004–2012) periods was 0.79 in children 0–4 years of age. In a separate report from Wales, the incidence of type 1 diabetes in children was modeled using a quadratic fit for trends over time [25]. The year with the highest incidence rate of type 1 diabetes in children 0–4 years was 2012, the year before the rotavirus vaccine was introduced and, afterwards, the incidence rate declined [25]. In a study from the northern UK (North East of England and North Cumbria), the incidence of type 1 diabetes in children (0–14 years) was reported from 2012 to 2020 [26]. The incidence rate of type 1 diabetes was greater in the pre-vaccine period (25.5 per 100,000 children in 2012) and decreased to 16.6 per 100,000 children in year 2020 (post-vaccine).

A study of children within the Clinical Practice Research Datalink in the United Kingdom was also conducted [27]. The authors reported that there was no association between rotavirus vaccination and childhood type 1 diabetes, using mixed-effects Cox regression. Their initial results showed that the percentage of children who developed type 1 diabetes in the vaccinated group was significantly lower than in the unvaccinated group (*p* = 0.001, as shown in their first table). The numbers, as reported, would yield a risk ratio of 0.76, indicating a 24% reduction (95% CI: 11%, 35%) in the risk of type 1 diabetes in the vaccinated children compared to the unvaccinated group [28]. In the final analysis as reported in the study, models with random general practice intercepts (with an average of <1 case per practice) were used, which yielded non-significant results due to low statistical power from inflated variance [27]. In addition, the inclusion of birth year (a surrogate for vaccination status) in the models contributed to multicollinearity which would bias the results towards the null. In a sensitivity analysis from this study using only children who were born after the introduction of the vaccine (i.e., with concurrent controls), the hazard ratio was 0.76 (vaccinated vs. unvaccinated) [27]. This is comparable with the results using raw numbers, suggesting a protective effect with vaccination.

In the Republic of Ireland, the monovalent rotavirus vaccine was introduced in December 2016 [29]. A study was conducted to evaluate the incidence of type 1 diabetes in children from 2014 to 2018 using the Irish Childhood Diabetes National Register [30]. Before the introduction of the rotavirus vaccine, the incidence of type 1 diabetes increased in children 0–4 years of age (annual percentage change = 37.5%, Figure 1); the rates increased from 13.61 to 18.71 per 100,000 per year [30]. After the vaccine was introduced, the incidence decreased (annual percentage change = −5.4%) from 13.25 to 12.53 per 100,000 per year. The risk ratio comparing the incidence rate after vaccination (12.53/100,000/year) to before vaccination (18.71/100,000/year) was 0.67.

### 2.4. Evidence Relevant to the Use of Both Pentavalent and Monovalent Vaccine

In Austria, rotavirus vaccination was introduced within the childhood vaccine schedule in 2007 [31]. Both monovalent and pentavalent vaccines were used at various points in time. Prior to the introduction of the rotavirus vaccine, the incidence of type 1 diabetes in children 0–4 years increased (annual percentage change = 7.1%) [31]. Afterwards, incidence rates decreased (annual percentage change = −0.9%; Figure 1). Graphical results indicated that the incidence rate of type 1 diabetes started to decline in 2008 (post-vaccine) in children of 2 years of age.

In Sweden, the introduction of the rotavirus vaccine (both monovalent and pentavalent) was gradual over time [32]. Two regions of the country introduced rotavirus vaccination for children in 2014. Other regions introduced the vaccine from 2016 to 2018 and, in September 2019, the vaccine became available in the remaining regions. Using information from the nationwide registry, the incidence of type 1 diabetes was evaluated from 2009 to 2019 (pre-vaccine to partial roll-out) [32]. When the data were stratified by geographic area, the results were not significant (incidence rate ratio = 0.86 in one area and 0.85 in another area). When the data were pooled for the regions with pre-post-vaccine information, the incidence rate ratio was 0.85 (*p* = 0.010) indicating that rotavirus vaccination yielded lower rates of type 1 diabetes in young children ages 0–4.9 years [28].

In Canada, publicly funded rotavirus immunization programs were initiated at different times across the provinces [33]. Age-specific data were available from British Columbia [34]. From years 1997 (pre-vaccine) to 2023 (post-vaccine), the annual percentage change in incidence rates of type 1 diabetes in children 1–4 years slightly decreased (−0.3% with linear fit). The monovalent rotavirus vaccine was introduced in January 2012 and the pentavalent vaccine was introduced in April of 2018 [33]. Within this analysis, the highest incidence rate of type 1 diabetes in children 1–4 years occurred in 2008, prior to vaccine introduction [34].

In the United States, data were available from a large cohort study with individual-level information regarding the types of vaccines used [20]. Children were followed from birth with continuous insurance coverage throughout the follow-up period to determine vaccine usage and disease diagnoses. The incidence of type 1 diabetes was 20.6 cases per 100,000 person-years in those who were not vaccinated for rotavirus. The incidence was lower (12.2 cases per 100,000 person-years) in children who completed the rotavirus vaccine series. Children who received the entire rotavirus vaccine series (either RotaTeq or Rotarix) had a 33% lower risk of developing type 1 diabetes (95% CI: 17%, 46%) compared to children who were not vaccinated [20]. The effect appeared to be specific to the rotavirus vaccine; the children who completed both the rotavirus and DTaP (i.e., diphtheria, tetanus and pertussis) vaccine series were 56% less likely to develop type 1 diabetes than children who completed just the DTaP series (95% CI: 46%, 64%). The results were robust to the sensitivity analyses (e.g., use of insulin, hospitalization for type 1 diabetes) and when using the historical comparison. From years 2001 (pre-vaccine) to 2017 (post-vaccine), there was a 3.4% decrease annually in the incidence rates of type 1 diabetes in children aged 0–4 years [20].

Other evidence from the United States provides insight [35]. Using population-based case ascertainment in five regions of the United States, the incidence of type 1 diabetes in children 0–4 years of age significantly decreased from 2003 (pre-rotavirus vaccine) to 2012 (post-rotavirus vaccine) [35]. The pentavalent vaccine was introduced in 2006 and the monovalent vaccine was introduced in 2008 [20].

An additional study from the United States included information from the Vaccine Safety Datalink [36]. Within their cohort, data regarding early-onset type 1 diabetes (8 months to 4 years) was available in a secondary analysis. The hazard ratio comparing the rotavirus-vaccinated versus the unvaccinated was 0.73 (0.39, 1.38). However, the study was underpowered due to a low number of unvaccinated children in the sample (power = 29%, alpha = 0.05, 2-tailed) [28]. In the entire sample, only 2.8% were unvaccinated [36]. The point estimate (0.73) suggests a possible protective effect, but the study was insufficiently powered to address this particular research question.

In summary, the country with the highest rates of childhood type 1 diabetes—Finland—exhibited the largest absolute reduction in early-onset type 1 diabetes after the pentavalent vaccine was introduced [28]. The risk ratios generated from the vaccination studies indicated a 21% to 37% reduction in the incidence of early-onset type 1 diabetes with the use of the pentavalent vaccine. Some countries, such as the Republic of Ireland, however, used the monovalent vaccine and the incidence of type 1 diabetes declined in the youngest children as well. Countries that recommended either monovalent or pentavalent rotavirus vaccines experienced a 15% to 33% lower risk of early-onset type 1 diabetes.

It is difficult to compare the relative effectiveness of the monovalent versus the pentavalent vaccine because circulating strains of rotavirus vary with location and time, and viral exposure may differ for children in one country versus another. In the United States, both monovalent and pentavalent vaccines were available at the same time and individual-level data regarding the types of vaccine given to each child were known [20]. The data suggested that RotaTeq may have a greater protective effect on rates of type 1 diabetes than Rotarix [20]. The hazard ratio was 0.63 (95% CI: 0.50, 0.78) for infants who were given the entire RotaTeq series and was 0.73 (95% CI: 0.50, 1.07) for infants who received the Rotarix series, when regressed simultaneously. The comparable hazard ratios were 0.66 (95% CI: 0.53, 0.81) and 0.90 (95% CI: 0.62, 1.31) when regressed in separate models [20]. In addition, for Mexico and Israel, the greatest reduction in incidence rates of type 1 diabetes came with the use of RotaTeq in their immunization schedules.

## 3. Critique of Epidemiologic Studies

**Figure 2:**
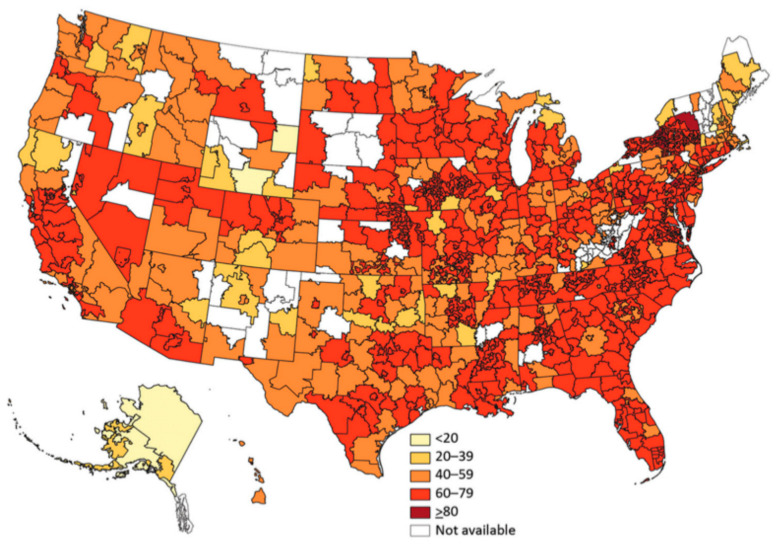
Percentage of infants (≤1 year of age) covered by private health insurance who completed the rotavirus vaccination series in the United States, 2010–2017. Source: *Emerging infectious diseases*, 25(10), 1993–1995 [39].

The studies with individual-level information on vaccination status for each child contained more precise information than those studies using group-level data. For the pre- versus post-vaccination comparisons using group-level data, the percentage of children who actually received the vaccine (vaccine coverage) may impact the results. There were some countries where rotavirus vaccine coverage was moderate [37]. In the United States, for example, rotavirus vaccine coverage depended upon a variety of factors including the type of vaccine series employed (Rotarix or RotaTeq) and the type of insurance available [38]. In a large cohort where individual-level data were available, vaccine coverage with the fully completed series (either Rotarix or RotaTeq) was 68.6% [39]. Coverage was quite variable across different areas of the country as shown in Figure 2.

Rotavirus vaccination rates tended to be lower in rural areas of the United States [39], where the incidence of type 1 diabetes was greater [40].

Early-onset type 1 diabetes is a rare condition and as such, the epidemiologic evidence highlights the importance of sample size. Some of the published studies were inadequately powered [36,41]. In one of the studies, the hazard ratio was 0.73 in children 0–4 years (95% CI: 0.39–1.38), comparing fully vaccinated versus unvaccinated [36]. The power to detect a significant difference was only 29% [28]. This particular database contained mainly children who were vaccinated (93.1% for the complete rotavirus series) and only a small percentage of unvaccinated children (2.8%) [36]. Statistical power not only depends upon the total number of children in the study, but the numbers of children with the outcome in each of the comparison groups.

In other epidemiologic studies, overadjustment in statistical models was problematic [16,27]. In one instance [16], the percentage of vaccinated children who developed type 1 diabetes was significantly lower than the percentage of unvaccinated children who developed type 1 diabetes (*p* = 0.002 for the difference) [17]. In the article, however, the results were not significant due to overadjustment; the model included adjustment for baseline rates of type 1 diabetes which were essentially a different representation of the dependent variable [16].

For studies in which data were collected over long periods of time (including both prior to and after the vaccine was introduced), adjusting for birth year was questionable. If almost all children in the earlier years were unvaccinated and almost all the children in more recent years were vaccinated, birth year is essentially a proxy for vaccination status. Inclusion of both vaccination status and birth year in statistical models can lead to biased non-significant results [27]. This can be rectified by modifying the study design (using concurrent controls) or altering the analyses.

Survival analysis was often used to compare outcomes in the cohort studies with individual-level data. An underlying assumption of Cox regression is that outcome rates in the comparator groups are proportional over time. In one study, this assumption was violated [42]. When this occurs, the analysis could be split into time periods to reflect the differences in the hazard ratios at specific periods, or an interaction term could be used. An extended model would yield multiple hazard ratios for the main comparison (vaccinated versus unvaccinated). Moreover, vaccination status was incorrectly modeled as time-varying rather than fixed [42]. At 12 months of age, each infant either received the entire vaccine series, was partially vaccinated, or was not vaccinated; this did not change after 12 months. When such irregularities in modeling occur, it is useful to examine the raw number of events in the comparator groups when these are available. In that study, the percentage of rotavirus-vaccinated children who developed type 1 diabetes was 0.0475%. The percentage of unvaccinated children who developed type 1 diabetes was 0.0603%. This difference was statistically significant (*p* = 0.006) [17], indicating that the incidence of type 1 diabetes was lower in the children who were vaccinated against rotavirus.

In longitudinal studies using birth cohorts, caution should be taken when evaluating the evidence for older children. In some studies, more than half the children were lost to follow-up by the age of 5 years, yet hazard ratios were calculated for the older children [36,43]. If investigators wish to explore the risk of type 1 diabetes in older children, the study design could involve assembling a cohort of children without type 1 diabetes who were school-aged (e.g., 6–7 years of age) with an observation period in the subsequent years. As such, the proper power calculations and similar follow-up periods for the comparator groups would enhance valid comparisons.

In studies of rotavirus infection and type 1 diabetes, exclusion of subjects based on their reasons for vaccination (or not undergoing vaccination) should be avoided. Biologically, the comparison is between those infected with rotavirus and those not infected. By willfully excluding people who were unvaccinated because these individuals were hesitant to vaccinate ignores important biologic pathways of the disease process. Hesitancy is not an independent risk factor of the outcome; it acts through its relationship to vaccination status. The reasons for not receiving the vaccine could be investigated in a different study and, indeed, there are studies that report these findings [44].

Table 1 below lists the epidemiologic studies included in this review with information regarding the type of vaccine, years covered, and study design.

## 4. Connecting Epidemiologic Evidence with Underlying Physiology

### 4.1. Timeline of Infection

**Figure 3:**
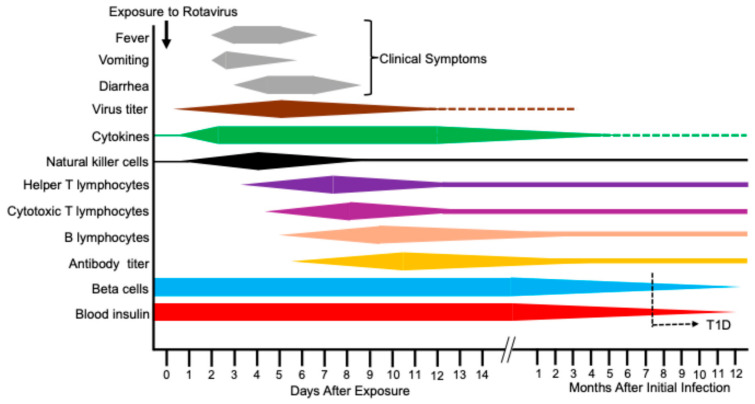
Timeline of rotavirus infection.

Following exposure to rotavirus, virions pass through the stomach and flow into the small intestine [2,45]. As virions infect intestinal enterocytes and increase in numbers, infected enterocytes release stress signals (e.g., cytokines) as part of the innate immune response. The increase in cytokines leads to fever and vomiting in the child, which typically begins 2–3 days after rotavirus exposure (Figure 3).

Circulating natural killer cells (NKs) recognize stress receptors on infected cells and create holes in the cell membranes, resulting in death of the infected cell. As infected cells lyse, holes in the enterocyte cell layer are created, allowing fluids from the internal portions of the small intestine tissues to flow into the gut lumen, causing diarrhea [45,46], which typically begins 3–4 days after exposure.

The cytokines and antigen presenting cells (APCs) signal T lymphocytes to begin differentiation and proliferation of virus-specific helper T (T_H_) lymphocytes, cytotoxic T (CT) cells, and B lymphocytes [47,48]. Some B cells secrete virus-specific antibodies throughout the body, while other B cells display the antibodies on their cell membranes as a signal to cells of infection. As responses of the immune system rise during days 4 through 10, virus titers decrease, as do the gastrointestinal symptoms. In some children, a reduction in beta cells and insulin is sufficient to impact blood glucose, which prompts initial clinical symptoms of type 1 diabetes.

### 4.2. Pathophysiology

There are several mechanisms through which the pancreatic beta cells may undergo damage with rotavirus infection. They include activation of autoimmune T cells, by molecular mimicry, through direct infection of the beta cells by the rotavirus, through Toll-like receptors, and via bystander activation. These processes are described below.

**Figure 4:**
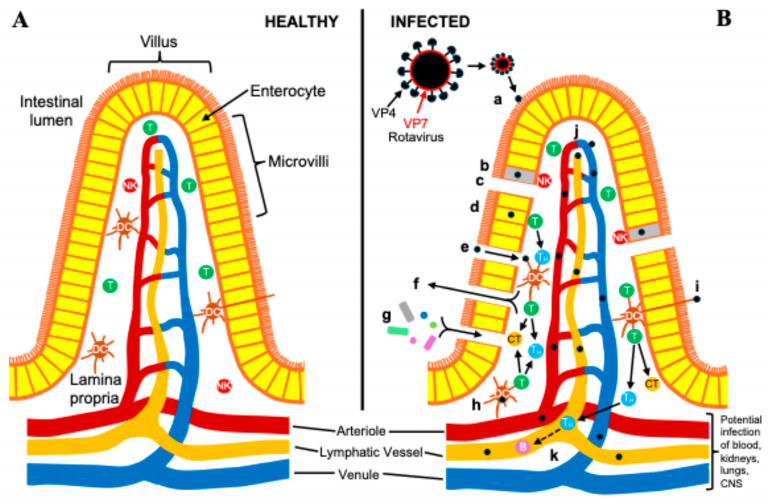
Progression of rotavirus infection. (**A**) The normal situation in the small intestine. (**B**) Infection of the intestinal villi by rotaviruses.

Rotavirus infection of enterocytes lining the small intestine is shown in Figure 4. Damage to intestinal villi by rotaviruses and the loss of epithelial integrity in the intestinal lining involve complex interactions among cell types. The immediate effect of rotavirus infection is attachment to enterocytes by viral capsid proteins VP4 and VP7, followed by entry into the enterocytes (Figure 4B, a) [46,49].

After entry into the cell, viral replication ensues. The infected enterocyte releases stress signals to its cell surface that are recognized by NK lymphocytes, thereby releasing chemicals (perforins and granzymes) that create holes in the cells, stimulating apoptosis (Figure 4B, b). The cell disintegrates causing a hole in the intestinal wall (Figure 4B, c), while other enterocytes become infected and display epitopes of the viral proteins on their major histocompatibility complex (MHC) cell receptors, becoming APCs. These are contacted by T lymphocytes which release cytokines to alert and stimulate T_H_ and CT lymphocytes, which begin replicating and differentiating to recognize, find, and destroy rotavirus virions (Figure 4B, d) [47,48]. Other rotavirus virions enter through the holes in the intestinal wall [46] and are contacted by dendritic cells (DCs), which secrete cytokines and display epitopes of rotavirus proteins on their cell surface receptors. This causes inflammation and signals immature T cells to mature into T_H_ and CT lymphocytes specific for rotavirus (Figure 4B, e) [48]. Holes in the enterocyte layer allow water, ions (primarily Na^+^ and Cl^−^), and other inclusions to leak out of the lamina propria and into the intestinal lumen producing diarrhea (Figure 4B, f). Holes in the enterocyte layer also allow other viruses, bacteria, and gut contents to enter the lamina propria, causing additional immune responses (Figure 4B, g). DCs can become infected by rotaviruses, inducing the secretion of cytokines indicating cell stress.

APCs can also signal T lymphocytes by direct contact (Figure 4B, h) [45,46,47,48,49]. Some DCs have cellular extensions that traverse the enterocyte layer and contact rotavirus virions that are within the intestinal lumen (Figure 4B, i). They secrete cytokines and process the virus particles into epitopes of the virus to act as APCs, signaling T lymphocytes within the lamina propria. This causes replication and differentiation into other T lymphocytes (e.g., CTs and T_HS_) (Figure 4B, i). Some virus particles can enter the lymphatic vessels, arterioles and venules (Figure 4B, j), leading to dissemination and/or infection of other tissues, such as blood, kidney, lung, heart, pancreas, and central nervous system (CNS) (Figure 4B, k) [45,50].

**Figure 5:**
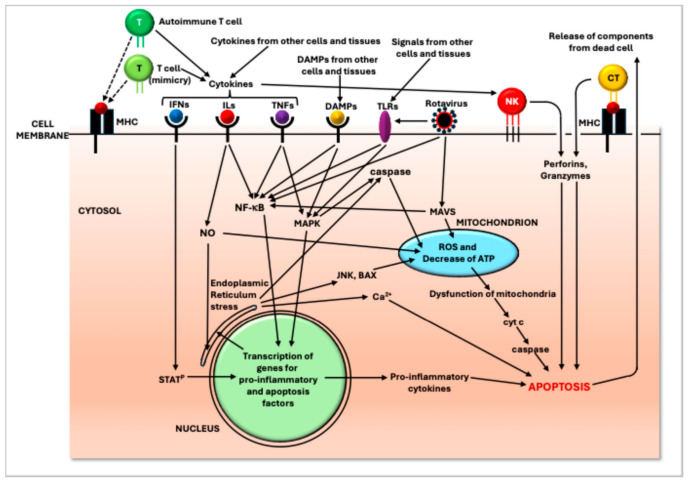
Mechanisms that kill pancreatic beta cells leading to type 1 diabetes.

During activation of T cells, several types of signals can affect beta cells, as indicated in Figure 5. Autoimmune lymphocytes may proliferate (Figure 5, upper left). The primary autoimmune T lymphocytes that are reactive towards pancreatic beta cells are specific for insulin autoantibodies (IAAs), islet cell cytoplasmic antigen (ICA), glutamic acid decarboxylase 65 kDa isoform (GAD65), zinc transporter 8 (ZnT8), and insulinoma antigen 2 (IA-2) [6,8,47,51,52,53]. These attach to MHC receptors displaying one or more epitopes projecting from the outer cell surface. The attached autoimmune T lymphocytes react as if a foreign antigen has been detected and they begin secreting cytokines that stimulate the beta cell to produce pro-inflammatory cytokines and initiate apoptosis.

Another mechanism for beta cell apoptosis is through mimicry [51,52,54,55]. IA-2 has a nine amino acid portion that is 100% similar (56% identity) to a short portion of rotavirus VP7 that might be sufficient for an anti-VP7 T cell to cross-react with an authentic IA-2 epitope being presented on MHC receptors on the beta cell surface. This results in secretion of cytokines that promote apoptosis of the beta cell.

When CT lymphocytes (Figure 5, upper right) attach to MHC receptor on a beta cell (either due to an autoimmune reaction or mimicry), they create holes in the cell membrane by secreting perforins and granzymes; in the process, they activate cell apoptosis systems within the cell. NK cells have receptors that dock with beta cells that have receptors indicating stress/infection on their surface. The beta cells may also present aberrant proteins, missing MHC type I receptors, or react to the presence of certain cytokines that may stimulate apoptosis. Similar to CT lymphocytes, NK cells secrete perforins and granzymes to disrupt the cell membrane and stimulate apoptosis of the beta cells.

Signal transduction systems (Figure 5, upper middle) include those responsive to cytokines and damage-associated molecular pattern signal molecules (DAMPs, including DNAs, ATP, and other cellular components) and are released by stressed, dying, or dead cells [56,57]. Receptors on beta cells respond to these via signaling pathways, including mitogen-activated protein kinases (MAPKs), to increase the production of pro-inflammatory cytokines and apoptosis-promoting factors. Interferons (IFNs) cause phosphorylation of signal transducer and activator of transcription (STAT) proteins that are translocated into the nucleus where they increase transcription of genes for pro-inflammatory cytokines and apoptosis-promoting proteins. Interleukins (ILs) cause increases in nitric oxide (NO) which promotes endoplasmic reticulum (ER) stress, leading to increases in caspase, c-Jun N-terminal kinase (JNK), bcl-2-like protein 4 (BAX, a kinase), and cytoplasmic Ca^2+^. Together, these cause dysfunction of mitochondria leading to increases in reactive oxygen species (ROS), reduction in ATP synthesis, and release of cytochrome c. All of these trigger cell apoptosis [58]. Interleukins (ILs) also cause phosphorylation of nuclear factor-kappa B (NF-κB), thus activating it as a transcription factor, which increases transcription of genes for pro-inflammatory cytokines, leading to apoptosis. Tumor necrosis factors (TNFs) also cause phosphorylation of NF-κB, which leads to production of pro-inflammatory cytokines and phosphorylation of MAPKs (including JNKs). In addition to increasing transcription of genes for pro-inflammatory cytokines, they can activate caspases that cause dysfunction of mitochondria. Both pathways lead to apoptosis of the cell [57].

Rotaviruses contain double-stranded RNA (dsRNA). Toll-like receptors (TLRs) are activated by contact with dsRNA [59]. TLRs are found on beta cells, as well as on DCs, T cells, and B cells. They induce the production of pro-inflammatory cytokines. Beta cells can react to direct contact with rotavirus virions through TLR3, which activates the NF-κB and MAPK pathways, leading to massive production of IFN-β and apoptosis of beta cells [59]. In addition, rotavirus infection can activate NF-κB and mitochondrial antiviral-signaling proteins (MAVS) pathways, causing apoptosis of beta cells. These pathways cause dysfunction of mitochondria in cells with contact of the virus, resulting in apoptosis [60]. They may also affect beta cells through the process of “bystander activation”, where cytokines secreted by DCs and T cells, activated by infection or cell stress, cause inflammation and apoptosis of pancreatic beta cells [61].

### 4.3. Vaccine Considerations

The epidemiologic evidence suggests that the pentavalent vaccine may provide greater protection against early-onset type 1 diabetes than the monovalent vaccine. The pentavalent vaccine (RotaTeq) contains VP7 of rotavirus serotype G3 which is associated with GAD65 and IA-2 mimicry [55]. The monovalent vaccine (Rotarix), however, lacks the association with IA-2. Thus, RotaTeq may provide broader neutralizing antibody protection [62].

The pentavalent vaccine (RotaTeq) contains 5 human-bovine live-attenuated reassortant strains of rotavirus while the monovalent vaccine (Rotarix) contains 1 human live-attenuated strain (G1P[8]) [11]. Of the 42 G (outer capsid glycoprotein VP7) genotypes of rotavirus and 58 P (outer capsid protease-sensitive protein VP4) genotypes, 14 G and 17 P types in 80 combinations have been reported in humans [2,63]. Six combinations (G1P[8], G2P[4], G3P[8], G4P[8], G9P[8], and G12P[8]) are common in humans worldwide, with the first four being the most frequent. In addition to the large number of genotypes, rotaviruses have a segmented genome consisting of 11 separate double-stranded RNAs [64]. When cells are infected with more than one strain, the resulting virus particles can contain reassorted RNA segments, creating novel variants. This situation is similar to that of influenza A viruses, which contain eight separate negative-sense RNAs present as their genomes; reassortant strains can result in novel combinations consisting of human, swine, and avian segments. Rotaviruses can also infect various mammalian hosts [65]. This situation complicates vaccine development for these viruses, not only due to rapid mutation rates, but also to the variability and potential rapid changes in genome composition due to reassortment of the RNA segments.

The genetic background of an individual may affect the susceptibility to rotavirus infection. Attachment by rotavirus isoforms P[4], P[6] and P[8] to human cells are dependent on histo-blood group antigens [66,67,68,69,70]. These antigens are controlled by several genes, including fucosyltransferase gene 3 (*FUT3*), fucosyltransferase gene 2 (*FUT2*), and *ABO* genes. FUT3 is responsible for adding a fucose molecule to an oligosaccharide and FUT2 causes secretion of the oligosaccharide to the outer surface of the cell. Individuals who are FUT2-positive (secretor) are at greater risk of P[4] and P[8] rotavirus infection [66,67,68,69,70]. Individuals who are FUT3 (Lewis)-negative have elevated risk of P[6] rotavirus infection, irrespective of secretor status [66]. Of relevance to the studies in this review, P[4] and P[8] rotavirus infections are common in Europe and North America (P[6] infection occurring in sub-Saharan Africa and Asia). RotaTeq and Rotarix both contain the P[8] genotype. Approximately 20% of individuals in Europe and North America are estimated to be non-secretors (non-secretors FUT2 polymorphism) and thus, at lower risk of rotavirus infection [66]. These differences in host susceptibility have been taken into account during vaccine development and relate to variations in vaccine efficacy in different populations. There have been small studies investigating the association between histo-blood group antigens and rotavirus “vaccine take” (host response to the vaccine measured through seroconversion or vaccine shedding), generally in high (childhood) mortality countries [66]. However, the degree to which this translates to robust measures showing effectiveness in preventing severe diarrhea and mortality in subpopulations has not yet been proven. It is important to note that both *FUT2* and *FUT3* are expressed in newborns at very low levels and only reach adult levels in years 2 and 3, which renders children especially susceptible to rotavirus infection in the first few years after birth [71].

Although Rotarix and RotaTeq have been evaluated in the medical literature for their association with type 1 diabetes, there are other rotavirus vaccines (Rotasiil, Rotavac, Rotavin-M1 in Vietnam, LLR in China) currently in use which were developed to prevent severe diarrhea in young children [11]. Randomized controlled trials have been conducted to evaluate vaccine efficacy in reducing severe diarrhea, as well as their safety. In the latest update, 60 trials were conducted with 228,233 children who were observed during the first two years of life [72]. During those first two years, Rotarix prevented 90% of cases of severe rotavirus diarrhea in low-mortality countries and 77% such cases in medium-mortality countries [72]. The comparable figures for RotaTeq were 96% and 79%. Countries that evaluated the incidence of early-onset type 1 diabetes in this review would be classified as low-mortality, except for Mexico which was classified as medium-mortality. The risk of intussusception occurred at the same frequency in children who received Rotarix as children who were given the placebo (risk ratio = 0.87, 95% CI: 0.52, 1.46) [72]. The risk of intussusception was also similar for RotaTeq versus placebo (risk ratio = 0.74, 95% CI: 0.38, 1.42) [72].

In addition to the six rotavirus vaccines in current use, there are 12 next-generation rotavirus vaccines under development [73]. The goal of such vaccines is to “improve the effectiveness and impact of rotavirus vaccination programs in low-income countries and lower middle-income countries, by further reducing morbidity and mortality associated with rotavirus infection and associated moderate-to-severe diarrhea” [73]. Childhood type 1 diabetes is less frequent in the low-income countries and the association between rotavirus vaccination and early-onset type 1 diabetes has not been reported in such countries. However, in some countries, such as India, the impact of rotavirus vaccination on early-onset type 1 diabetes should be explored because of its large population of young children (more than 113 million children <5 years of age) [74].

Population-level vaccination strategies are specific to the countries in which they are adopted, given the circulating strains of rotavirus, population characteristics, and vaccines available. For the countries with low childhood mortality and higher rates of childhood type 1 diabetes (such as those included in this review), the decision to vaccinate for rotavirus is likely centered on its efficacy in preventing severe diarrhea—not on the likelihood of preventing early-onset type 1 diabetes. However, this added benefit may be helpful in reaching an overall decision of risk versus benefit.

## 5. Discussion

Worldwide, gastroenteritis from rotavirus infection is the principal cause of diarrheal deaths in infants [75]. It has a short incubation period and is highly infectious, being readily transmitted through stool (with 10^11^ virus particles/gram) [75]. There is currently no antiviral treatment for this disease; only supportive care is available after symptoms begin. Lowering the burden of this disease involves prevention through vaccination. Data from randomized controlled trials revealed that rotavirus vaccines prevented severe diarrhea in young children [72]. The data from this review suggests that rotavirus vaccination may also reduce the likelihood of ancillary pancreatic damage, resulting in beta cell apoptosis and the onset of type 1 diabetes. Yet, completion of the rotavirus vaccine series (globally) was 56% in 2023 and 59% in 2024 [76]. In Europe (the region with the highest incidence rates of childhood type 1 diabetes), rotavirus vaccine coverage was 42% in 2024 [76]. In the United States, there have been recent declines in completion of the rotavirus vaccine series [77]. This is expected to have a considerable impact on hospitalizations for severe gastroenteritis in young children and additional cases of early-onset type 1 diabetes. It is important to note that there are no recommended “catch-up” vaccinations for this virus after 8 months of age. Therefore, if the vaccine was not administered during the appropriate times, it would be a missed opportunity for prevention.

The evidence for the association between rotavirus infection and early-onset type 1 diabetes emanates from several factors:

- Rotavirus infection is the most frequent gastrointestinal infection in infants and young children [1]. It was within this group of young children (<5 years of age) that the association between rotavirus infection and type 1 diabetes was found. Both affect the same types of individuals.
- Rotaviruses can directly infect pancreatic beta cells, the lack of which is the underlying cause of type 1 diabetes [7].
- The incidence of type 1 diabetes in young children decreased after the introduction of rotavirus vaccination [10,14,15,17,18,19,20,22,24,28,30,31,35]. This pattern was apparent across a number of countries. This pattern was evident even though the introduction of the rotavirus vaccine occurred in different years in the countries.
- Experiments demonstrate that rotavirus infection decreases plasma insulin and increases blood glucose in animals [8].
- Multiple studies have reported significant associations between rotavirus infection and increased titers of pancreatic islet autoantibodies in children [6,53,78,79,80,81], supporting the hypothesis that the rotavirus infection may induce type 1 diabetes.
- The association between the vaccine and lower rates of type 1 diabetes appeared to be specific to the rotavirus vaccine in young children. Those children who completed both the rotavirus and DTaP vaccine series were 56% less likely to develop type 1 diabetes than children who completed just the DTaP series [20].
- There was some evidence to suggest that the pentavalent rotavirus vaccine may offer more protection against type 1 diabetes than the monovalent vaccine. Individual-level data from a large cohort of infants who were observed longitudinally showed that those who received the pentavalent vaccine series were 37% less likely to develop type 1 diabetes [20]. The results were not significant for infants receiving the monovalent vaccine series [20]. The pentavalent vaccine contains VP7 of rotavirus serotype G3 which is associated with GAD65 and IA-2 mimicry [55]. The monovalent vaccine lacks the association with IA-2. Thus, the pentavalent vaccine may provide broader neutralizing antibody protection [62].
- Pancreatic beta cell destruction has been shown to occur after rotavirus infection through multiple mechanisms. These include activation of autoimmune T cells, molecular mimicry, through Toll-like receptors, and by bystander activation.
- Meta-analyses found similar results. In one study, vaccinated children <5 years of age had a lower risk of developing type 1 diabetes compared to those who were not vaccinated (relative risk = 0.84, 95% CI: 0.75–0.95) [82]. In another study, the pooled hazard ratio was 0.87 (95% CI: 0.78–0.98), indicating a 13% lower risk of type 1 diabetes in vaccinated children compared to unvaccinated children [83].
- The most widely recognized risk factor for childhood type 1 diabetes is a family history of the disease. The population-based studies in which the comparison was within an entire country (pre- versus post-vaccination) show that, when family history is held constant, lower incidence rates of type 1 diabetes occurred after vaccination was introduced [10,14,15,18,19,22,24,28,30,31,35]. These associations were not due to confounding by family history [28].

Other studies contribute to the evaluation of this association. Breastfeeding provides passive immunity to rotavirus infection in infants [84]. Some, but not all, studies have shown an association between breastfeeding and type 1 diabetes [85]. It is possible that some of the protection of breastfeeding may be due to the passive immunity to rotavirus infection.

There has been interest in investigating possible nutritional risk factors for type 1 diabetes. A large international randomized controlled trial demonstrated that infants weaned to cow’s milk formula had the same incidence of type 1 diabetes as infants weaned to a hydrolyzed casein-based formula, suggesting that cow’s milk was not a risk factor for type 1 diabetes [86]. These results indicate that the ingestion of cow’s milk is not likely to be a potential confounder for the association between rotavirus infection and early-onset type 1 diabetes because it is not related to the outcome. Rotavirus infection, however, does alter the gut microbiome and therefore, may play a role in the biologic pathways as indicated in Section 4.2.

Viral triggers for the onset of type 1 diabetes have been widely studied and there is evidence that different viruses could potentially play a role in its etiology [87]. It is possible that some of the underlying inflammatory gastrointestinal effects of viral infection may be similar to what is experienced during rotavirus infection. Enteroviruses such as enterovirus B, enterovirus C, coxsackievirus B1, and coxsackievirus B4 have been shown to elevate the risk of type 1 diabetes [88,89]. Some evidence also implicates SARS-CoV-2 as a contributor to the incidence of type 1 diabetes over the past decade [90,91,92]. Both congenital rubella infection and congenital cytomegalovirus infection were strongly associated with the development of early-onset type 1 diabetes [93]; in this study, none of the infants with congenital infections who received the entire rotavirus vaccine series later developed type 1 diabetes. However, in the group that did not receive rotavirus vaccine, four developed diabetes (1 of 32 with rubella and 3 of 224 with cytomegalovirus) [93]. This may suggest that repeated viral exposures enhance the risk of early-onset type 1 diabetes.

The hygiene hypothesis is sometimes discussed in reference to childhood vaccinations [94]. This hypothesis posited that exposure to microorganisms in early childhood enables the development of the immune system and may result in fewer health-related problems later on. The scientific literature does show that early exposure to certain bacteria in the gastrointestinal tract may be advantageous, indicating beneficial effects of a healthy microbiome [95]. This phenomenon is different than early exposure to known viral pathogens. Based on the present evidence, the hygiene hypothesis is not true for rotavirus. Infection due to rotavirus commonly occurs in very young children and can result in severe diarrhea, hospitalization, and death [2,5]. Vaccination during infancy reduces these sequelae in children. An additional benefit of rotavirus vaccination has been the reduction in seizures in young children [4,96]. We could find no evidence that willful exposure to rotavirus in early childhood provides a benefit. In fact, there is evidence that vaccination against rotavirus infection is beneficial in neonates [97].

## 6. Recommendations

There are several topics for further scientific investigation. Many etiologic studies of type 1 diabetes were conducted in children who were at higher risk due to family history of the disease or the presence of genetic markers. Because monozygotic twins are not always concordant for the diagnosis of type 1 diabetes [98], studies are warranted which examine the risks of type 1 diabetes in children without a family history. From information published as a supplement in one investigation [36], data were available for children who did not have a parent or older sibling with type 1 diabetes. The incidence of type 1 diabetes in the rotavirus-vaccinated group was 0.0996% and the incidence in the unvaccinated was 0.2062% (a 2-fold difference). The results are suggestive for further investigation.

Another research approach would be to widen the “at risk” group. Individuals with type 1 diabetes are known to have elevated rates of other autoimmune diseases [99]. If children are selected for risk status based on a family history of autoimmune thyroid disease, celiac disease, rheumatoid arthritis, and/or other autoimmune diseases, this could potentially increase the positive predictive value of finding those children at risk of developing type 1 diabetes. Investigations could assess whether the predictors of type 1 diabetes were similar (or different) than the group solely defined by family history of type 1 diabetes.

While it is important to investigate diarrheal diseases in children in low- and middle-income countries because of the enormous impact on morbidity and mortality [100], it is also prudent to remember that diarrheal diseases, such as rotavirus infection, still affect many children in high-income countries [101]. This is particularly important because of the changing recommendations regarding rotavirus vaccination in the United States and the increase in vaccine hesitancy [102]. Prior to the advent of rotavirus vaccination, community-acquired rotavirus infection in high-income countries primarily occurred in previously healthy children [5]. Of the countries mentioned in this report, the United States had the greatest number of young children (18 million 0–4 years of age in 2025) [74]. Mexico had the next greatest, with 10 million young children in 2025 [74]. The United Kingdom was next in line with 3.5 million, Canada with 1.9 million, and Australia with 1.5 million children 0–4 years of age in 2025 [74]. Rotavirus vaccine coverage may have a substantial public health impact in these countries because of the numbers of children involved. Investigations from other locations would be welcome.

The studies in this review indicate that there is an attenuation in the incidence of early-onset type 1 diabetes after rotavirus vaccination. At this time, it is unknown whether this reflects long-lasting prevention or a delay in the onset of type 1 diabetes. Additional longitudinal studies are necessary to evaluate such questions. Early-onset type 1 diabetes is a life-threatening disease which shortens one’s lifespan and increases the risks of developing other chronic diseases such as cardiovascular disease, retinopathy, kidney disease, and neuropathies. Additional longitudinal research could delineate whether vaccination would reduce the population burden of these ensuant chronic diseases.

## 7. Conclusions

The evidence indicates that rotavirus infection at an early age increases a child’s risk of developing type 1 diabetes. It is a contributing factor in some children. Not all children with rotavirus infection develop type 1 diabetes; in fact, most do not. There are other factors involved in the etiology of early-onset type 1 diabetes. However, this does not negate the findings that rotavirus infection is likely to be one of the contributors to the development of this disease.

Rotavirus vaccination has been shown to dramatically reduce the number of hospitalizations and deaths due to acute gastroenteritis. It may also reduce the likelihood of pancreatic damage in some children, resulting in type 1 diabetes. Rotavirus vaccination not only prevents serious infection but can decrease the possibility of long-term sequelae which profoundly impact the daily lives of children.

## Figures and Tables

**Table 1:** Studies on the Incidence of Type 1 diabetes in children <5 years of age.

Country	Vaccine Type	Years in Study	Study Design	References
Australia	Rotarix or RotaTeq	2000–2015	Interrupted time-series. Pre- versus post-vaccine	[10]
Austria	Rotarix or RotaTeq	1989–2017	Time trends in incidence. Pre- versus post-vaccine	[31]
Canada (British Columbia)	Rotarix or RotaTeq	1997–2023	Time trends in incidence	[34]
Finland	RotaTeq	1995–2015	Time trends in incidence. Pre- versus post-vaccine	[15]
Finland	RotaTeq	2001–2015	Survey nested within a randomized trial	[41]
Finland	RotaTeq	2003–2018	Time trends in incidence	[14]
Finland	RotaTeq	2009–2014	Population-based cohort study	[16]
Ireland	Rotarix	2014–2018	Time trends in incidence	[30]
Israel	Rotarix or RotaTeq (2007–2010); RotaTeq (2011–2018)	2000–2016	Interrupted time-series. Pre- versus post-vaccine	[18]
Israel	Rotarix or RotaTeq (2007–2010); RotaTeq (2011–2018)	2002–2018	Interrupted time-series. Pre- versus post-vaccine	[19]
Mexico	Rotarix (2007–2011); RotaTeq (2011–2018)	2000–2018	Time trends in incidence	[22]
Sweden	Rotarix or RotaTeq	2009–2019	Time trends in incidence. Pre- versus post-vaccine	[32]
United Kingdom (Wales)	Rotarix	1990–2019	Time trends in incidence	[25]
United Kingdom	Rotarix	1994–2022	Time trends in incidence	[24]
United Kingdom	Rotarix	2010–2020	Cohort study	[27]
United States	Rotarix or RotaTeq	2001–2017	Cohort study	[20]
United States	Rotarix or RotaTeq	2002–2012	Time trends in incidence	[35]
United States	Rotarix or RotaTeq	2006–2017	Cohort study	[36]
United States	Rotarix or RotaTeq	2006–2017	Cohort study	[42]

## Data Availability

This review used information from published scientific studies, as referenced.

## Abbreviations

The following abbreviations are used in this manuscript:

AAPC	Average annual percentage change
APCs	Antigen presenting cells
BAX	bcl-2-like protein 4
CNS	Central nervous system
CT	Cytotoxic T
DAMPs	Damage-associated molecular pattern signal molecules
DCs	Dendritic cells
DTaP	Diphtheria, tetanus and pertussis
FUT2	Fucosyltransferase gene 2
FUT3	Fucosyltransferase gene 3
GAD65	Glutamic acid decarboxylase 65 kDa isoform
IAAs	Insulin autoantibodies
IA-2	Insulinoma antigen 2
ICA	Islet cell cytoplasmic antigen
IFNs	Interferons
ILs	Interleukins
JNK	c-Jun N-terminal kinase
MAPKs	Mitogen-activating protein kinases
MAVS	Mitochondrial antiviral-signaling proteins
MHC	Major histocompatibility complex
NF-κB	Nuclear factor-kappa B
NK	Natural killer
NO	Nitric oxide
ROS	Reactive oxygen species
STAT	Signal transducer and activator of transcription
T_H_	Helper T lymphocytes
TLRs	Toll-like receptors
TNFs	Tumor necrosis factors
UK	United Kingdom
ZnT8	Zinc transporter 8
